# Measurement properties of the Swedish version of the anterior cruciate ligament return to sport after injury scale (ACL‐RSI): A Rasch analysis

**DOI:** 10.1002/jeo2.12059

**Published:** 2024-06-12

**Authors:** Ramana Piussi, Sara Alenius, Kate E. Webster, Roland Thomeé, Albert Westergren, Peter Hagell, Eric Hamrin Senorski

**Affiliations:** ^1^ Sportrehab Sports Medicine Clinic Gothenburg Sweden; ^2^ Department of Health and Rehabilitation, Unit of Physiotherapy, Institute of Neuroscience and Physiology, Sahlgrenska Academy University of Gothenburg Gothenburg Sweden; ^3^ Sahlgrenska Sports Medicine Center Sahlgrenska Academy Gothenburg Sweden; ^4^ The PRO‐CARE Group, Faculty of Health Sciences Kristianstad University Kristianstad Sweden; ^5^ School of Allied Health, Human Services and Sport La Trobe University Bundoora Australia; ^6^ The Research Platform for Collaboration for Health, Faculty of Health Sciences Kristianstad University Kristianstad Sweden

**Keywords:** anterior cruciate ligament, knee, psychometric, reconstruction

## Abstract

**Purpose:**

This study aimed to investigate the measurement properties, according to the Rasch Measurement Theory, of the anterior cruciate ligament return to sport after injury scale (ACL‐RSI) in patients treated with ACL reconstruction in Sweden.

**Methods:**

ACL‐RSI responses from 1065 patients treated with ACL reconstruction were extracted from a rehabilitation‐specific registry. Rasch analyses were performed on ACL‐RSI item response data using the RUMM2030plus software. The analyses focused on targeting (person‐item threshold distribution), item hierarchy, response category thresholds, overall and individual item and person fit, differential item functioning (DIF), unidimensionality and reliability.

**Results:**

The ACL‐RSI had compromised fit to the Rasch model, including DIF and malfunctioning response categories. Several items correlate with each other and the presumptions to aggregate item responses into one single score were not met. When accounting for local dependency, the measurement properties of the ACL‐RSI improved in terms of model fit and DIF and unidimensionality were supported.

**Conclusion:**

The ACL‐RSI was found to have signs of multidimensionality and local dependency, that is, the answers to one item are influenced by the answers to other items. As such, researchers should be careful when using the ACL‐RSI as one single score to evaluate patients treated with ACL reconstruction, unless local dependency is accounted for in the scoring process.

**Level of Evidence:**

Level III.

**Abbreviations**
ACL: anterior cruciate ligament
ACL‐RSI: anterior cruciate ligament return to sport after injury scale
CI: confidence interval
CTT: classic test theory
CV: critical value
DIF: differential item functioning
IRT: item response theory
PCA: principal component analysis
PRO: patient‐reported outcome measure
PSI: Person Separation Index
RMT: Rasch measurement theory
RTS: return to sport
SD: standard deviation
SE: standard error
VAS: visual analogue scale

## INTRODUCTION

A commonly used patient‐reported outcome measure (PRO) to evaluate patients after anterior cruciate ligament (ACL) injury and reconstruction is the ACL return to sport after injury scale (ACL‐RSI), which was developed in 2008 and aims to assess athletes' emotions, confidence and risk appraisal related to return to sport (RTS) [27].

Items of the ACL‐RSI were developed through an extensive literature search, where five items related to fear, tension and nervousness were generated and included in an ‘emotions’ domain, five items were generated and included in a ‘confidence’ domain and two items were generated and included in a ‘risk appraisal’ domain [27]. The scale was found to have a Cronbach's *⍺* of 0.96, and inter item correlations had a mean of 0.69 (min–max, 0.49–0.83). Principal components analysis showed the presence of one underlying factor with an eigenvalue of 8.14 that accounted for 67.8% of the total variance. Individual scores for the 12 items were therefore summed, averaged and recalculated to provide a single on a 0–100 scale. Accordingly, the ACL‐RSI has always been reported as a single total score, considered by the research community to reflect ‘readiness to RTS’. Notably, readiness to RTS was not defined in the original paper in 2008. A systematic review from 2018 reported strong evidence for internal consistency, reliability and structural validity of the ACL‐RSI, whereas evidence for test–retest reliability, cross‐cultural validity, hypothesis testing validity and content validity was moderate or limited [9].

Psychometric evaluations of PROs can be divided into modern and classic test theory (CTT) [24]. Within CTT, tests such as Cronbach *⍺*, principal component analysis (PCA) or multitrait scaling analysis are employed, while item response theory (IRT) and Rasch measurement theory (RMT) represent modern test theory, of which RMT is considered superior [11, 22]. RMT, a specific form of IRT, is particularly distinguished by its ability to convert qualitative observations into measurable, linear data. Developed by Georg Rasch, this model is grounded in the fundamental measurement principles used in the physical sciences [11, 22]. Rasch posited that a mathematical model could be formulated to define stringent requirements for item‐level data, ensuring that the derived scales are not merely ordinal but represent true interval measures. This is achieved by calibrating each item based on its difficulty and by simultaneously evaluating the ability of the respondents, thereby creating a more precise and individualised measure of latent traits.

All published studies on the psychometric properties of the ACL‐RSI have been based on CTT [9], but modern test theory approaches are warranted for PROs [12]. The use of the Rasch model for the assessment of PROs in patients who suffer an ACL injury can be an important piece in building evidence of PROs psychometric properties, as the work by Comins et al. [8] has shown on the Knee injury and Osteoarthritis Outcome Score. Rasch analysis on the ACL‐RSI is still lacking. Therefore, this study aimed to investigate the measurement properties, according to RMT, of the ACL‐RSI in patients treated with ACL reconstruction in Sweden.

## METHOD

The present study's prospectively collected data were extracted from a rehabilitation outcome registry, Project ACL. The registry was established in 2014 and aims to improve the care of patients who suffer an ACL injury. Data consist of results from muscle function tests and PROs collected prospectively before the surgery (in case of ACL reconstruction) and after 10 weeks, 4, 8, 12, 18 and 24 months and every 5 years with ACL injury/reconstruction as a baseline [4, 10]. Prior to participation in Project ACL, written consent was collected. Ethical approval was obtained from the Swedish Ethical Review Authority (registration number: 2020‐02501).

### ACL‐RSI

Each of the 12 ACL‐RSI items is graded from 1 to 10, where 10 represents the best possible psychological response to RTS (highest confidence and emotion and lowest risk appraisal). The final score is calculated by summing item scores (highest score 120), and then converting the score to a 0–100 scale [25]. The ACL‐RSI has been translated into several different languages, including Swedish (used in this study) [14]. Table 1 presents an overview of items of the ACL‐RSI.

**Table 1:** Overview of items in the ACL return to sport after injury scale.

Item	Domain
1. Are you confident that you can perform at your previous level of sport participation?	Confidence
2. Do you think you are likely to reinjure your knee by participating in your sport?	Risk appraisal
3. Are you nervous about playing your sport?	Emotions
4. Are you confident that your knee will not give way by playing your sport?	Confidence
5. Are you confident that you could play your sport without concern for your knee?	Confidence
6. Do you find it frustrating to have to consider your knee with respect to your sport?	Emotions
7. Are you fearful of reinjuring your knee by playing your sport?	Emotions
8. Are you confident about your knee holding up under pressure?	Confidence
9. Are you afraid of accidentally injuring your knee by playing your sport?	Emotions
10. Do thoughts of having to go through surgery and rehabilitation again prevent you from playing your sport?	Risk appraisal
11. Are you confident about your ability to perform well at your sport?	Confidence
12. Do you feel relaxed about playing your sport?	Emotions

Abbreviation: ACL, anterior cruciate ligament.

### Sample

Patients aged 16–50 at the time of ACL reconstruction and registered in Project ACL with one ACL injury, treated with reconstruction were eligible for inclusion. Patients who did not respond to all ACL‐RSI items at the 12‐month follow‐up were excluded.

Since rehabilitation after ACL injury treated with reconstruction typically varies between 6 and 13 months [15] after surgery, demographic data and data from the ACL‐RSI from the 12‐month follow‐up were extracted from Project ACL.

**Figure 1:**
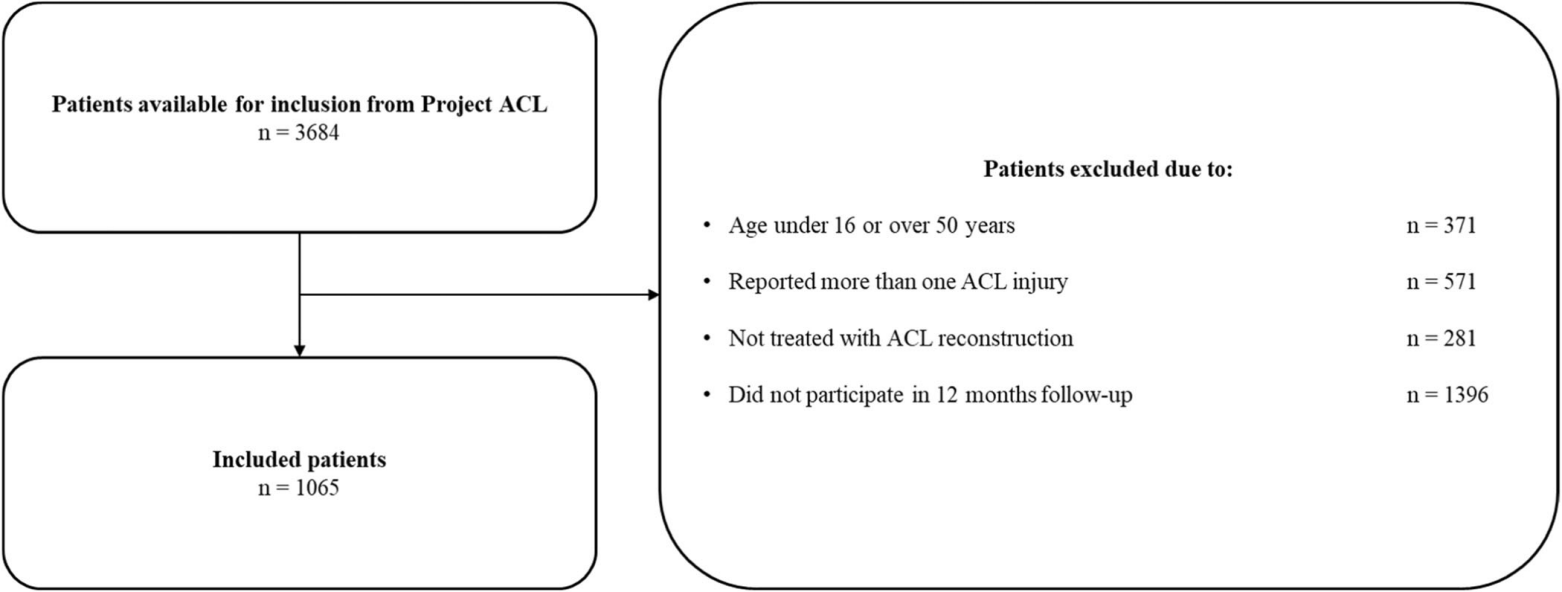
Flowchart of inclusion. ACL, anterior cruciate ligament.

ACL‐RSI responses from a total of 1065 patients were available from Project ACL. All patients answered all items. Figure 1 presents a flowchart of the inclusion process. There were 600 women (56%) and the mean age for the 1065 patients was 29.7 ± 8.7 years (Table 2).

**Table 2:** Demographics of included patients.

	Total	Women	Men
Sex, *n* (%)	1065	600 (56.3%)	465 (43.7%)
Age (years) mean (SD)	29.7 (8.7)	28.6 (8.9)	31.1 (8.2)
Height (cm) mean (SD)	173.9 (8.9)	168.3 (6.1)	181.2 (6.5)
Weight (kg) mean (SD)	72.3 (12.4)	65.6 (9.3)	80.9 (10.6)
BMI mean (SD)	23.7 (2.9)	23.1 (2.9)	24.6 (2.7)
Tegner preinjury level, *n* (%)			
1–5	164 (15.3%)	106 (17.6%)	58 (12.4%)
6	86 (8.1%)	58 (9.7%)	28 (6%)
7	177 (16.6%)	86 (14.3%)	91 (19.6%)
8	205 (19.2%)	132 (22%)	73 (15.7%)
9	299 (28.1%)	149 (24.8%)	150 (32.3%)
10	134 (12.6%)	69 (11.5%)	65 (14%)
Graft choice, *n* (%)			
Hamstring	790 (74.2%)	444 (74%)	346 (74.4%)
Patella	186 (17.4%)	104 (17.3%)	82 (17.6%)
Other	20 (2)	12 (2%)	8 (1.7%)
Missing	69 (6.4%)	40 (6.7%)	29 (6.2%)

Abbreviations: BMI, body mass index; *N*, number; SD, standard deviation.

### Analysis

Demographics for included patients were reported as numbers (*n*) and percentages (%) and mean and standard deviations (SDs). RMT analyses were performed on ACL‐RSI item response data using the RUMM2030plus software (RUMM Laboratory Pty Ltd.) [2]. In the analyses, response categories 1–10 were rescored to 0–9. In this study, the ACL‐RSI was analysed according to the unrestricted polytomous (‘partial credit’) Rasch model [3]. The analyses focused on targeting (person‐item threshold distribution) item hierarchy, response category thresholds, overall and individual item and person fit, differential item functioning (DIF), unidimensionality and reliability. Bonferroni adjustments for multiple null hypothesis testing were applied, with the *⍺* level of significance set at *p* < 0.05 [5].

### Targeting

Targeting illustrates the scale's ability to cover the range of the latent trait relative to the locations of the persons. A mean person location of <±0.5 logits has been suggested to indicate good targeting [13].

Reliability was estimated using the Person Separation Index (PSI) [1].

### Response category thresholds

Response category thresholds represent the points where there is an equal probability of responding in either of two adjacent response categories. Disordered thresholds signal that response categories do not work as intended.

### Item hierarchy

Patients with high scores on the ACL‐RSI are expected to have high probabilities of endorsing responses representing positive emotions, high levels of confidence and low levels of risk appraisal. The logic of the hierarchical order of item locations was considered to assess internal, content and construct validity.

### Overall and individual item and person fit; DIF

Overall fit can be assessed by the mean person and item standardised fit residuals. In a well‐fitting scale, mean standardised fit residuals should be close to 0, with an SD close to 1. Residuals between ±2.5 are generally considered acceptable [3]. Large positive item fit residuals suggest multidimensionality, while large negative item fit residuals suggest local dependence between items in the scale. In the context of the Rasch model, local dependency refers to a situation where responses to items on a questionnaire are not independent of each other. Local dependency may be due to multidimensionality or that the response to one item affects response(s) to other item(s).

DIF is an additional aspect of model fit that can be defined as ‘a difference in item performance between two comparable groups, i.e., groups that are matched with respect to the construct being measured by the test’ [7]. In this study, we tested for DIF by sex and age to explore whether items function in the same way between men and women and between younger and older patients. Age was dichotomised into older (29–50 years old) and younger (16–28 years old) according to the median age of the included patients.

### Unidimensionality

Unidimensionality refers to items representing only one dimension, and consequently inferring that the items can be summarised into a total score.

An additional analysis was performed where items were grouped conceptually: items 1, 4, 5, 8 and 11 were considered to represent ‘confidence’, items 3, 6, 7, 9 and 12 ‘emotion’ and items 2 and 10 ‘risk appraisal’.

## RESULTS

Analyses were conducted in three steps, presented in Table 3.

**Table 3:** Analyses.

Analysis	Specification	Rationale	Findings that differ from the main analysis
1	Complete analysis		– See results
2	Subtest of items according to construct at scale conception: emotion, confidence, risk appraisal	To address local dependency	– No positive correlations between residuals – Risk appraisal items present DIF for gender – Three underlying factors in PCA – Subtest analysis supports unidimensionality
3 (Supporting Information S1: Appendix)	Remove misfits: individuals with person fit residuals over 2.5	Exclude individuals with unexpected answer pattern	– Item 12 showed a misfit – DIF by gender in item 6

Abbreviations: DIF, differential item functioning; PCA, principal component analysis.

### Targeting

**Figure 2:**
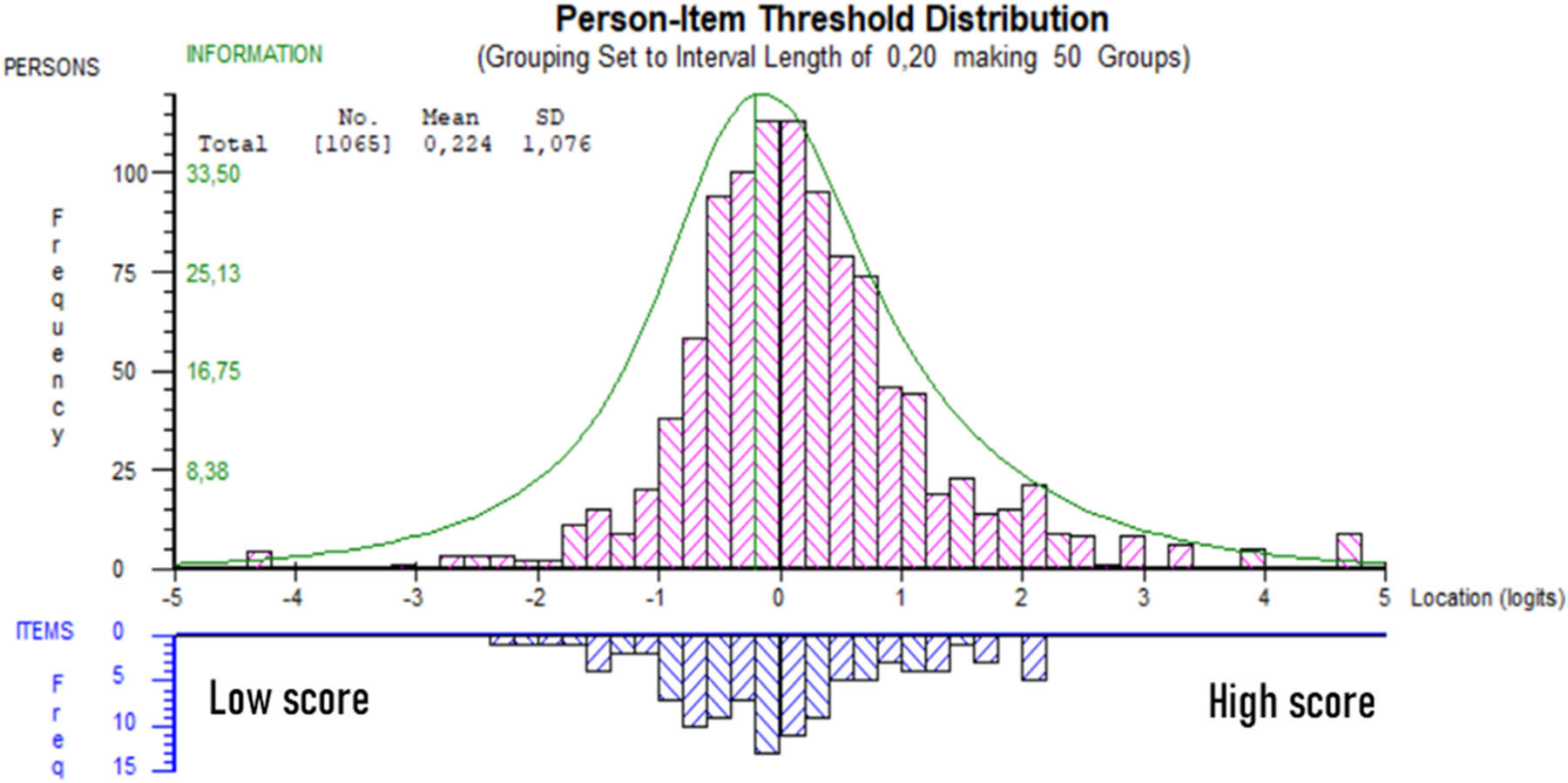
Person‐item threshold distribution, distribution of people (upper panel) and response category thresholds (lower panel) on the common logit metric (*x* axis; positive values = more positive emotions, confidence and risk appraisal).

The ACL‐RSI did not exhibit any floor or ceiling effect and the mean (SD) person location was 0.224 (1.08), suggesting adequate targeting (Figure 2). This provided good basic premises to evaluate the ACL‐RSI.

Estimations of reliability showed a PSI value of 0.941 and an *⍺* of 0.951, indicating good reliability of the ACL‐RSI.

### Response category thresholds

Items 6 (frustrated about considering your knee; panel A) and 10 (thoughts of surgery and rehabilitation; panel B) showed disordered thresholds (Supporting Information S1: Appendix Figure 1).

### Item hierarchy

The sorting of items by location order indicated that item 6 (frustrated about considering your knee) contained the highest representation of what the ACL‐RSI aims to measure, while item 1 (confident that you can perform at your previous level of sport participation) contained the lowest representation (Table 4). Items intended to assess confidence represented lower levels of the construct, whereas items intended to assess emotions represented higher levels of the construct.

**Table 4:** Item hierarchy.

Item	Description	Location (SE)	Domain	Interpretation
1	Confident to perform as before	−0.292 (0.019)	Confidence	Less confidence, negative emotions, high‐risk appraisal
8	Confident in your knee	−0.275 (0.021)	Confidence	
10	Go through the process again	−0.216 (0.017)	Risk appraisal	
11	Confident to perform well	−0.210 (0.020)	Confidence	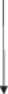
3	Nervous about playing	−0.208 (0.020)	Emotions	
4	Confident knee will not give away	−0‐054 (0.020)	Confidence	
2	Likelihood of reinjure	−0.053 (0.022)	Risk appraisal	
12	Relaxed about playing	−0.047 (0.020)	Emotions	
5	Confident to play without concerns	0.188 (0.019)	Confidence	
7	Not fearful of reinjury by playing	0.314 (0.020)	Emotions	More confidence, positive emotions, low‐risk appraisal
9	Not afraid of accidental reinjury	0.345 (0.020)	Emotions	
6	Not frustrating to consider the knee	0.509 (0.018)	Emotions	

*Note*: Item locations are expressed in logit values and represent the mean of each item's response category threshold locations. Negative locations represent less confidence, emotion and risk appraisal (i.e., the level of the measured construct) and positive locations represent more confidence, emotion and risk appraisal. Under ‘description’, a shortened version of the question was reported, to logically assess the order.

### Overall and individual item and person fit; DIF

Overall fit assessment showed a misfit to the Rasch model [total‐item *χ* ^2^ = 564.9, *p* < 0.0001, item mean (SD) fit residual = 1.18 (6.6) and person mean (SD) fit residual = −0.42 (1.6)].

Analysis of fit residuals showed that only items 8 (confident your knee is holding) and 12 (relaxed about playing your sport) did not signal misfit. To limit the potential effect of type 1 error, the sample size was adjusted to 500 individuals. Table 5 presents *p* values adjusted for sample size. For items 2 (are you likely to reinjure), 4 (confident your knee will not give way), 7 (not fearful of reinjury) and 8 (confident your knee is holding), the adjusted sample size altered the *p* values from significant to nonsignificant. Items 7 (not fearful of reinjury) and 9 (fear of accidental reinjury) showed the strongest residual correlation (0.464). Further residual correlations above the CV were observed between items 1 and 11 (0.187), items 4 and 5 (0.289), items 4 and 8 (0.176) and items 11 and 12 (0.286), indicating local dependency. To handle local dependencies, analysis number two was performed (Table 2).

**Table 5:** Fit residuals for each item.

Item	Fit residual	*χ* ^2^	*p* Value	*p* Value (*n* = 500)
1	3.821	7.504	0.585	0.937
8	−0.487	27.822	0.001	0.152
10	8.438	88.220	<0.001	<0.001
11	−4.613	38.619	<0.001	0.031
3	−5.432	60.178	<0.001	<0.001
4	−3.726	29.185	0.001	0.126
2	8.191	28.801	0.001	0.133
12	−1.012	44.262	<0.001	0.012
5	−6.792	56.952	<0.001	0.001
7	−2.935	21.382	0.011	0.337
9	4.388	10.603	0.304	0.830
6	14.363	151.389	<0.001	<0.001

*Note*: Fit residuals should be comprised between ±2.5. Items are sorted by location. Item locations are expressed in logit values and represent the mean of each item's response category threshold locations. *p* value adj = *p* Value adjusted for size, where size was reduced to 500 individuals.

Two items showed DIF by sex, item 9 (fear of accidental reinjury) and 10 (thoughts of surgery and rehabilitation) (Supporting Information S1: Appendix Figure 2A,B). Men were more likely to score high on fear of accidental reinjury (item 9). Women were more likely to score high for item 10 (thoughts of surgery and rehabilitation). Two items (7 and 11) showed DIF by age, where younger patients were more likely to be less confident to be able to perform at the preinjury level and to fear suffering a reinjury by participating in sports (Supporting Information S1: Appendix Figure 3A,B).

### Unidimensionality

In a test of unidimensionality, PCA identified two major factors on which items were loaded. Based on loading on each factor, items were grouped into two groups: items 1, 4, 5, 8, 11 and 12 in group 1 and items 2, 3, 6, 7, 9 and 10 in group 2. Then, separate person measures from these two item groups were compared on a person‐by‐person basis using *t* tests. This showed significantly different locations for 11% (Agresti–Coull 95% confidence interval [CI]: 9.2–13) of individuals, indicating multidimensionality.

#### Analysis 2: Subtest analysis

Since residual correlations and PCA showed a trend toward cluster items according to their conceptual representations, subtests were created to reflect the three conceptual domains. Thus, items 1, 4, 5, 8 and 11 (‘confidence’), items 3, 6, 7, 9 and 12 (‘emotions’) and items 2 and 10 (‘risk appraisal’) were combined into three corresponding subtests.

Reliability estimates in the subtest analysis were lower (PSI, 0.901; coefficient *⍺*, 0.856), compared to the item‐level analysis (0.941 and 0.951, respectively).

### Items hierarchy

Targeting showed a mean (SD) person location of 0.165 (0.646), which indicated good targeting. No floor or ceiling effects were present. Table 6 shows subtest locations.

**Table 6:** Hierarchy of subtests with conceptually grouped items.

Subtest	Location (SE)
Confidence (1; 4; 5; 8; 11)	−0.091 (0.012)
Emotions (3; 6; 7; 9; 12)	−0.069 (0.007)
Risk appraisal (2; 10)	0.160 (0.007)

*Note*: Item locations are expressed in logit values and represent the mean of each subtest's response category threshold locations. Negative locations represent less confidence, emotion and risk appraisal (i.e., the level of the measured construct) and positive locations represent more confidence, emotion and risk appraisal.

In agreement with the general pattern in Analysis 1, ‘confidence’ had the lowest location, whereas ‘risk appraisal’ now had the highest location. It is therefore easier for patients to respond in higher categories to confidence items than to risk appraisal items.

### Overall and individual item and person fit; DIF

Assessment of fit residual showed that confidence and risk appraisal subtests have large but nonsignificant fit residuals (Table 7).

**Table 7:** Fit residuals for each item.

Item	Fit residual	*χ* ^2^	*p* Value	*p* Value adj
Confidence (1; 4; 5; 8; 11)	4.565	8.360	0.786	0.977
Emotions (3; 6; 7; 9; 12)	−1.701	5.520	0.531	0.923
Risk appraisal (2; 10)	3.063	8.023	0.498	0.913

*Note*: Fit residuals should comprise between ±2.5 Items sorted by location. Item locations are expressed in logit values and represent the mean of each item's response category threshold locations. *p* value adj = *p* Value adjusted for size, where size was reduced to 500 individuals.

There was no DIF present by age; however, the risk appraisal subtest exhibited DIF by sex, where men systematically scored lower risk appraisal compared to women (Supporting Information S1: Appendix Figure 4).

### Unidimensionality

Unidimensionality was tested by comparing individual person locations based on the respective subtests; *t* tests showed significantly different person locations for 2.2%–3% (upper Agresti‐Coull 95% CI: 3.3%–4%) of the persons, suggesting unidimensionality. This is in accordance with the *c* (0.36), *r* (0.88) and *A* (0.9) indices, where a low value for *c* and high values for *r* and *A* suggests unidimensionality.

## DISCUSSION

This is the first comprehensive analysis of the ACL‐RSI based on RMT, demostrating that the ACL‐RSI has good reliability and targeting. However, the analysis revealed limitations due to the correlation between several items and the unmet assumptions necessary to aggregate item responses into one single score. In addition, the ACL‐RSI had compromised fit to the Rasch model, including DIF and malfunctioning response categories. When accounting for local dependency, the measurement properties of the ACL‐RSI improved in terms of model fit, and DIF and unidimensionality were supported. The aim of a Rasch analysis can be expressed as to assess and diagnose how a rating scale functions. In our analysis, the local dependency needed to be addressed for the ACL‐RSI to respect traits for unidimensionality, and consequently, our results provide information on how to improve the ACL‐RSI so it can be used as one single score.

Targeting of the ACL‐RSI was good with no floor or ceiling effects, thus the ACL‐RSI appears to cover the full range of the latent trait. While two items showed disordered thresholds, the other items had ordered thresholds. For the items with disordered thresholds, it is difficult for respondents to distinguish between 10 unspecified levels, which suggests that the ACL‐RSI may benefit from considering fewer and more distinct response categories, preferably including category labels [21].

The hierarchical arrangement of ACL‐RSI items indicates that items related to ‘confidence’ are at lower levels, while ‘emotions’ like fear and frustration are at higher, more favourable levels, with ‘risk appraisal’ items in between. The clinical interpretation would be that it appears easier for patients to achieve high confidence than to overcome negative emotions like fear, which suggests that high confidence is necessary before fear of reinjury can be addressed. Fear of reinjury has been reported in the literature as the main reason for not returning to sport in patients after ACL injury [6, 19]. When local dependency was addressed, the subtest hierarchy placed risk appraisal highest and confidence lowest. There was no overlap between the locations of item groups when standard errors were considered. Thus, it is more likely for patients to rate that they have high confidence, but less likely to rate that thoughts of surgery and rehabilitation again prevent them from playing, which can be considered clinically logical. Nevertheless, we did not control for whether patients in our sample had returned to sport or not.

Our results indicate local dependency within the ACL‐RSI, where responses to certain items influence others, or an additional variable impacts response. This local dependency, which includes both response and trait dependencies, skews reliability estimates [17]. Originally, the ACL‐RSI's reliability was overestimated at 0.951; addressing local dependency by merging items into subtests lowered this to a more accurate 0.856. Notably, items with similar content, like fear of reinjury (items 7 and 9) and confidence in sports performance (items 1 and 11), showed significant residual correlations due to their overlap in wording, influencing the scale's apparent unidimensionality [17]. Item 7 reads ‘Are you fearful of reinjuring your knee by playing your sport?’ and item 9 reads ‘Are you afraid of accidentally injuring your knee by playing your sport?’. While one could argue that no injury is non‐accidental in nature, the difference between the items is subtle, thus, items correlate.

The ACL‐RSI, that was originally developed to measure athletes' emotions, confidence and risk appraisal post‐ACL reconstruction [27], has recently been tied to ‘readiness to RTS’. However, this concept was not specified in its initial design, leading to potential issues in reflecting a singular construct. Addressing local dependency supported the scale's intended unidimensionality, despite the earlier lack of a defined measurement framework for its key constructs.

In terms of DIF, younger patients exhibited lower confidence and greater fear of reinjury, impacting their scores on the ACL‐RSI compared to older patients. This aligns with studies showing that emotional responses significantly affect younger athletes' psychological state towards RTS after ACL reconstruction [18, 23, 26].

### Strengths, limitations and future directions

We included over 1000 patients in this study, which is sufficiently large for a Rasch analysis [16]. The population was heterogenous for sex and age, the two variables used in the DIF analysis. However, we did not control several confounding factors that may have affected how a patient responds to the ACL‐RSI, which include RTS, type of sport, preinjury activity level and concomitant injuries. A further limitation was related to the chosen time of follow‐up: we chose to include responses 12 months after ACL reconstruction as this is a common time for RTS. It is possible that results from the analysis would be somewhat different at other follow‐ups, such as 8 or 24 months after ACL reconstruction. Despite this limitation, we believe that the sample clinically represents the variation of patients treated with ACL reconstruction. Collectively, our findings are robust, however, should be generalised cautiously.

The ACL‐RSI was initially designed to measure athletes' emotions, confidence and risk appraisal. Future refinements should consider a nuanced approach, potentially treating the PRO as unidimensional after addressing local dependencies and grouping items according to domains. To consider fewer and more distinct response categories, possibly with category labels, could possibly enhance the ACL‐RSI's ability to differentiate between individuals who vary in the trait, ability, attitude or condition that the questionnaire is intended to measure. Notably, a short version of the ACL‐RSI was developed [25]. Efforts to perform a Rasch analysis on the short version should be made to evaluate the short version's properties.

### Clinical implications

Several risks can be identified with the clinical use of the Swedish version of the ACL‐RSI. First, the ACL‐RSI is assessed as one single score, but because of how the questions relate to each other and cover different emotional aspects, this score might not fully represent how patients feel about RTS. One practical example is that patients can be deemed as ready for RTS, as shown by results on PROs, but actually did not feel ready to do so, which has been reported in the literature [20]. Second, the Swedish version of the scale assessed in this study has 10 levels for answering questions, but patients might have a hard time choosing the right level that matches how they feel, which can make their answers less accurate. Finally, the scale does not work the same for everyone. Depending on factors, such as age and sex, the scale might give different results, thus clinical decisions based on the ACL‐RSI scores may be less accurate for certain populations.

### Conclusion

The ACL‐RSI was found to have signs of multidimensionality and local dependency, that is, the answers to one item are influenced by the answers to other items. As such, researchers should be careful when using the ACL‐RSI as one single score to evaluate patients treated with ACL reconstruction, unless local dependency is accounted for in the scoring process. Based on our results, there is a good opportunity to enhance and improve the ACL‐RSI to be used as one single score.

## AUTHOR CONTRIBUTIONS

Ramana Piussi, Sara Alenius, Albert Westergren and Peter Hagell were primarily responsible for data analysis and interpretation. Ramana Piussi, Albert Westergren, Peter Hagell and Eric Hamrin Senorski were responsible for drawing and writing the manuscript. Kate E Webster and Roland Thomeé made important contributions to the writing and finalisation of the manuscript.

## CONFLICT OF INTEREST STATEMENT

The authors declare no conflict of interest.

## ETHICS STATEMENT

Ethical approval was obtained from the Swedish Ethical Review Authority (registration number: 2020‐02501).

## Supporting information

Supplementary information.

## Data Availability

The data sets used and/or analysed during the current study are available from the corresponding author upon reasonable request.
