# Supplementary material for: Measurement properties of the Swedish version of the anterior cruciate ligament return to sport after injury scale (ACL‐RSI): A Rasch analysis
Source: J Exp Orthop. 2024 Jun 12;11(3):e12059. doi: 10.1002/jeo2.12059 (PMC11167408; doi:10.1002/jeo2.12059)
Supplement: Supplementary file 1 — Supplementary information. [file JEO2-11-e12059-s001.docx]

***Appendix for Rasch analysis of the ACL-RSI***

**Detailed method description:**

***Targeting***

Targeting, that is, person-items threshold distribution, illustrates the scale´s ability to cover the range of the latent trait relative to the locations of the persons. The scale ability is illustrated by comparing the location of persons and items on the same logit scale. A mean person location of < ±0.5 logits has been suggested to indicate good targeting.[18]

Reliability was estimated using the Person Separation Index (PSI), which is conceptually similar to coefficient alpha.[2] In addition, since there were no missing responses in the data, coefficient alpha was also estimated.

***Response category thresholds***

Response category thresholds represent the points where there is equal probability of responding in either of two adjacent response categories. As such, they indicate whether response categories function as intended. Disordered thresholds signal that response categories do not work as intended, which may be due to, e.g., that respondents are not able to distinguish between categories.

***Item hierarchy***

Patients with high scores on the ACL-RSI are expected to have high probabilities to endorse responses representing positive emotions and high levels of confidence and low levels of risk appraisal. Conversely, patients with low scores on the ACL-RSI are expected to have low probabilities to endorse responses representing positive emotions, low levels of confidence and high levels of risk appraisal. This is the foundation for the location of items and persons on the latent logit continuum and allows to examine how the construct varies as a patient moves from lower to higher levels of emotion, confidence, and risk appraisal and how this is in accord with clinical and theoretical experiences. The logic of the hierarchical order of item locations was considered to assess internal content and construct validity.

***Overall and individual item and person fit; differential item functioning***

Overall fit can be assessed by the mean person and item standardized fit residuals. In a well-fitting scale, mean standardized fit residuals should be close to 0, with a standard deviation close to 1. Standardized fit residuals represent the difference between observed and expected scores, and differences between expected and observed responses are tested by an approximate Chi-square statistic, which should be nonsignificant to support model fit. However, the Chi-square can increase with large sample size. One way to account for this type 1 error is to algebraically adjust the sample size in the analysis, not affecting other aspects of data such as locations or fit residuals.[16] Accordingly, a sensitivity analysis with an adjusted sample size to n = 500 was performed.[13] Fit was also tested at the individual item and person level using the approximate Chi-square statistic as well as standardized fit residuals. Residuals between ±2.5 are generally considered acceptable.[4] Large positive item fit residuals suggest multidimensionality, while large negative item fit residuals suggest local dependence between items in the scale. In the context of the Rasch model, local dependency refers to a situation where responses to items on a questionnaire are not independent of each other. Local dependency, therefore, violates this assumption and occurs when the responses to some items are directly influenced by responses to other items beyond the influence of the underlying trait being measured. Local dependency may be due to multidimensionality or that the response to one item affects response(s) to other item(s). Local dependency can affect various aspects of the measurement process and yields artificially high reliability indices. Local dependency was further explored by examining item residual correlations, expressed as relative correlations. A critical value (CV) of 0.12 (based on the 99^th^ percentile, chosen to perform a robust analysis) as determined using a web based application based on the method suggested by Christensen et al.[8] (<http://publicifsv.sund.ku.dk/~kach/Q3/critical_values_Yens_Q3.html>) was calculated.

Differential item functioning (DIF) is an additional aspect of model fit that according to Clauser[9] can be defined as “a difference in item performance between two comparable groups, i.e., groups that are matched with respect to the construct being measured by the test”. A DIF factor should not be a factor that is comprised within the construct the scale aims to measure.[14] In this study, we tested for DIF by sex and age to explore whether items function in the same way between men and women and between younger and older patients. Age was dichotomized into older (29–50 years old) and younger (16–28 years old) according to the median.

***Unidimensionality***

Unidimensionality refers to items representing only one dimension, and consequently inferring that the items can be summarized into a total score. Unidimensionality is a part of construct validity, which refers to whether the instrument assesses what it is aimed to assess. For the assessment of unidimensionality, the approach applied consisted of the identification of two item sets from a PCA of residuals. Then, separate person measures based on the two item sets were estimated and compared on a person-by-person basis using t-tests. The number of cases that differ significantly (p <0.05) were then determined. If ≤5% of tests are significant, or the lower bound of a binomial 95% confidence interval (CI) of the observed proportion overlaps 5%, then it has been suggested that unidimensionality can be inferred.[12] In this study, the binomial 95% CI was calculated according to Agresti and Coull.[1]

An additional analysis was performed where items were grouped conceptually: items 1, 4, 5, 8, and 11 were considered to represent “confidence”, items 3, 6, 7, 9 and 12 “emotion”, and items 2 and 10 “risk appraisal”. This was done for two related reasons. First, items within each group were combined into a single “larger” item (subtest) representing that conceptual group, thus yielding a scale consisting of 3 subtests in the analysis. This absorbed any local dependency within each item group. The change in the reliability estimate (compared to the initial item level analysis) indicates the degree of dependency, and the indices c r and A can be used to address dimensionality. A describes the non-error variance common to all subtests, c characterizes the variance that is unique to the subtests (relative to the common variance = 1), and r is the latent correlation between the subtests. A subtest analysis performed on an approximate unidimensional scale will return high values for both A and r, and a low value for c.[4, 37] Specifically, an A value over 0.9 is indicative of a unidimensional model.[31] Second, the t-test procedure described above was applied to “confidence” and “emotion” items, that is, individual person measures were compared based on whether they were derived from items representing “confidence” or “emotion”.

**Results appendix:**

| A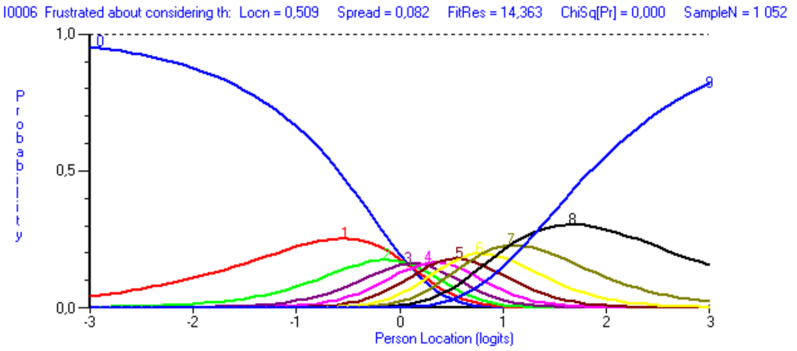 | B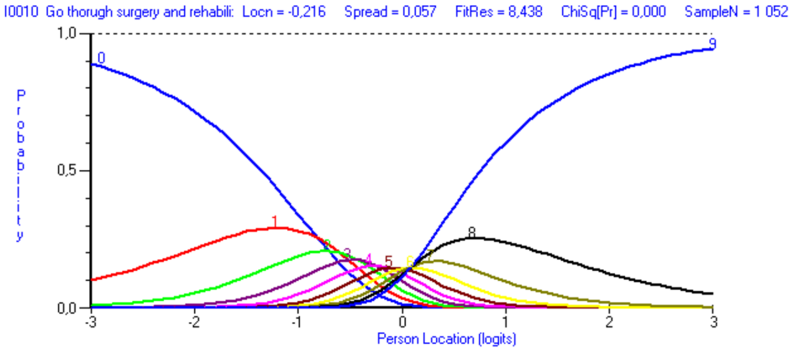 |
| --- | --- |
| C  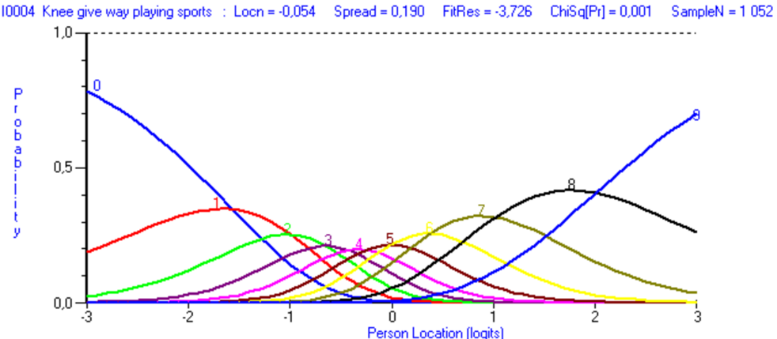 | D  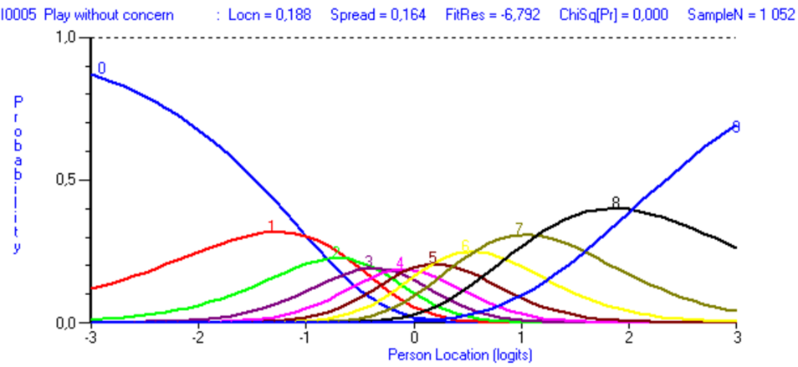 |

Appendix Figure 1: Disordered thresholds in items 6 (A) and 10 (B). For comparison, Panels C and D represent two items (4 and 5, respectively) with ordered thresholds. Each coloured line represent the probability (y-axis) of responding in that response category at various locations on the latent variable (x-axis). Disordered thresholds mean that the response category never is the most probable response.

| A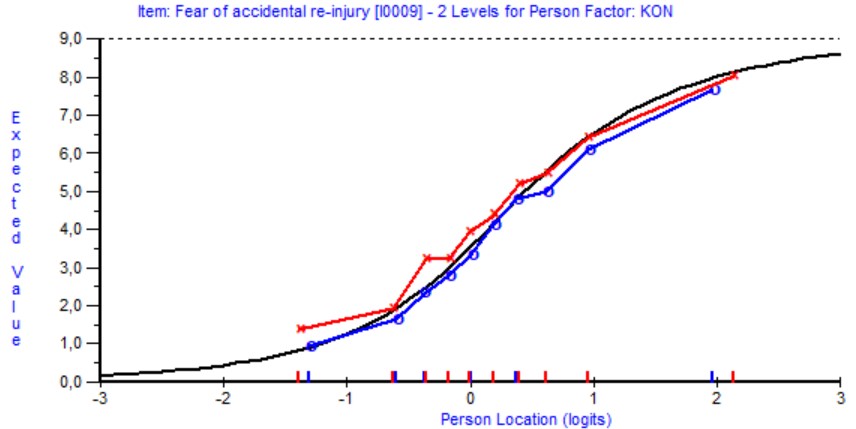 | B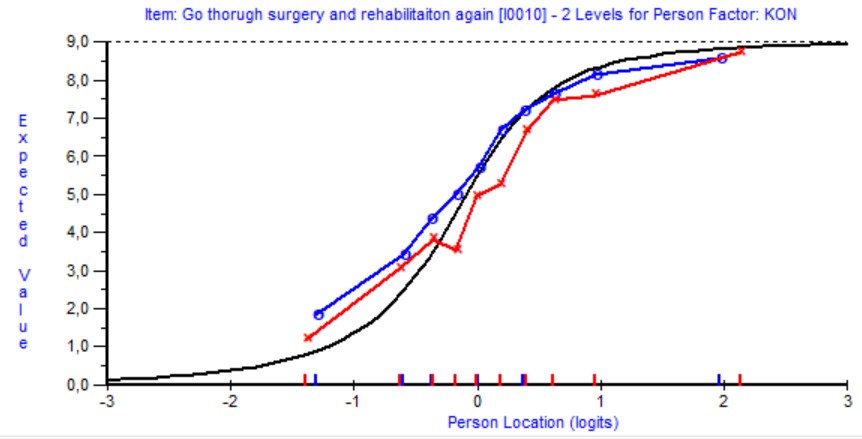 |
| --- | --- |

Appendix Figure 2A and 2B: Differential item functioning (DIF) between women (blue lines) and men (red lines) for items 9 (A) and 10 (B).

| A 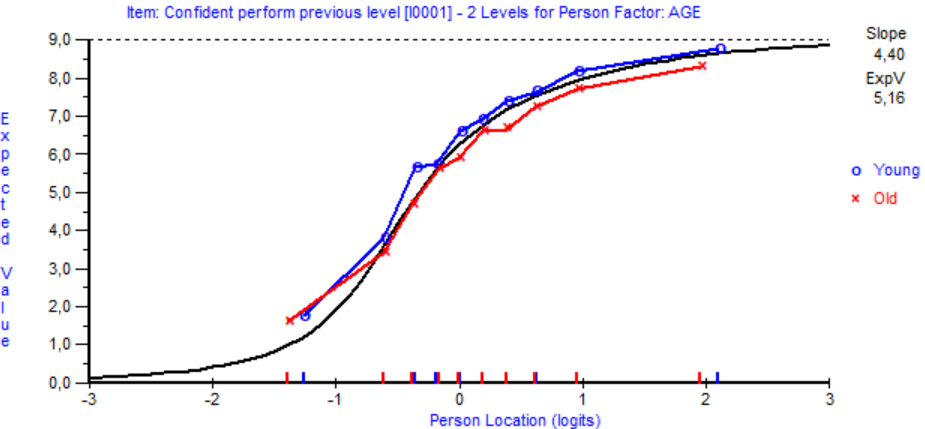 | B 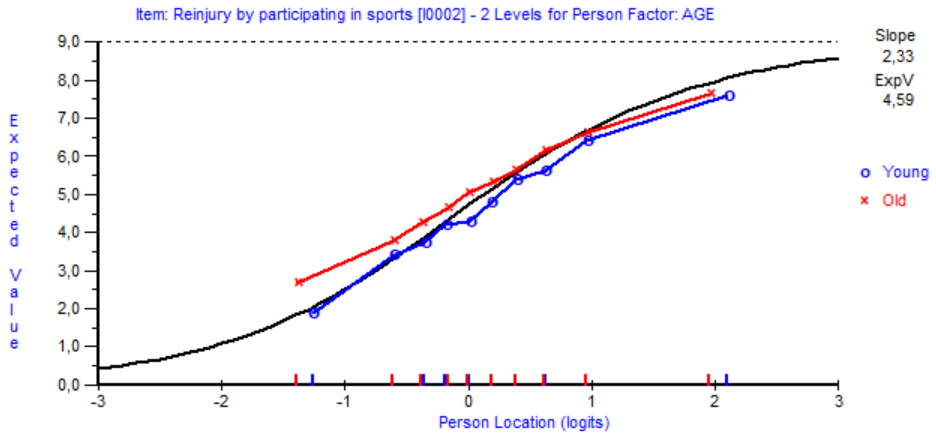 |
| --- | --- |

Appendix Figure 3A and 3B: Differential item functioning (DIF) between older (blue lines) and younger (red lines) patients for items 1 (A) and 2 (B).


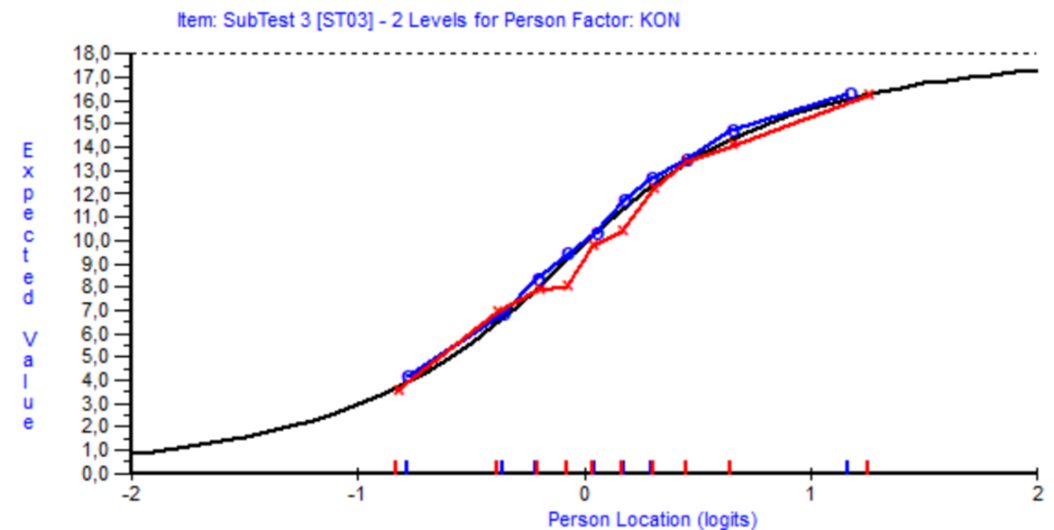


Appendix Figure 4: Differential items functioning (DIF) between women (blue lines) and men (red lines) for the risk appraisal subtest.

Assessment of person fit showed that there were 167 (15%) individuals with fit residuals outside the ±2.5 range (4% >2.5 and 11% <-2.5).

***Analysis 3: Excluding misfitting individuals***

Excluding misfitting persons did not alter results in a meaningful way (data not shown). For overall fit assessment still showed misfit: individual item fit improved with only item 8 showing misfit, but one additional item (item 6) showed DIF by sex. Adjusting sample size to n=500 improved overall fit (although Chi Square was still significant), and no item showed misfit.
